# Exploring the Epidemiology and Survival Trends in Pediatric Major Salivary Gland Malignancies: Insights from the National Cancer Database

**DOI:** 10.3390/curroncol30070456

**Published:** 2023-06-25

**Authors:** Madison Coleman, Jia Liang, Jeffrey C. Rastatter, Rebecca S. Arch, Jessica Gartrell, Daniel C. Chelius, Anthony Sheyn, Cai Li, Celine Richard

**Affiliations:** 1 Department of Otolaryngology, University of Tennessee Health Science Center College of Medicine, Memphis, TN 38103, USA; 2 Department of Pediatric Otolaryngology, Le Bonheur Children’s Hospital, Memphis, TN 38103, USA; 3 Division of Pediatric Otolaryngology, St. Jude Children’s Research Hospital, Memphis, TN 38103, USA; 4 Department of Biostatistics, St. Jude Children’s Research Hospital, Memphis, TN 38103, USA; 5 Division of Pediatric Otolaryngology–Head and Neck Surgery, Ann & Robert H. Lurie Children’s Hospital of Chicago, Chicago, IL 60611, USA; jrastatter@luriechildrens.org (J.C.R.);; 6 Department of Otolaryngology-Head and Neck Surgery, Northwestern University Feinberg School of Medicine, Chicago, IL 60611, USA; 7 Department of Oncology, St. Jude Children’s Research Hospital, Memphis, TN 38105, USA; jessica.gartrell@stjude.org; 8 Department of Otolaryngology-Head and Neck Surgery, Pediatric Head and Neck Tumor Program, Baylor College of Medicine, Texas Children’s Hospital, Houston, TX 77030, USA

**Keywords:** major salivary gland cancers, National Cancer Database, mucoepidermoid carcinoma, acinic cell adenocarcinoma, histology, age, gender, race, negative margins, 5-year survival rates

## Abstract

Objective: To investigate the clinicopathological, therapeutic, and survival data on pediatric major salivary gland cancers. Materials and Methods: National Cancer Database (NCDB) query from 2004 to 2018. Results: In total, 967 cases of individuals under the age of 21 were identified. Most cancers affected the parotid gland (86%). Mucoepidermoid carcinoma (41.3%) and acinic cell adenocarcinoma (33.6%) were the most common. Tumors occurred more often from age 11 to 21, and females were more affected. Histology varied by age, gender, and race. In the 0–5 age group, mucoepidermoid carcinoma and myoepithelial carcinoma/sarcoma/rhabdomyosarcoma were the most common pathologies. In patients over 5 years old, mucoepidermoid carcinoma was the most frequent tumor in boys, while acinic cell adenocarcinoma was more common in girls. African American patients had a higher incidence of mucoepidermoid carcinoma, while White patients in the 0–5 age group had a higher incidence of myoepithelial carcinoma/sarcoma/rhabdomyosarcoma tumors. Low-grade tumors were commonly diagnosed at stage I, but the 0–5 age group had a high frequency of stage IV tumors. The overall 5-year survival rate was 94.9%, with 90% for the 0–5 years age group and 96% for the 11–15 years age group. Negative margins were associated with higher 5-year survival rates in high-stage tumors (93%) compared to positive margins (80%). Submandibular malignancies had worse 5-year survival rates across all age groups. Conclusions: Major salivary gland malignancies in pediatric patients exhibit variations in histopathologic characteristics by age, gender, and race. Negative margins impact 5-year survival rates, especially in high-stage tumors.

## 1. Introduction

Pediatric salivary gland neoplasms are the fourth most common pediatric head and neck tumors after those found in the nasopharynx, skin, and thyroid. They account for 8.1% of all pediatric head and neck tumors and can occur at any age, but their incidence increases with age [1,2]. The majority of these tumors are found in the parotid gland, followed by the submandibular glands. Minor salivary gland neoplasms are rare in children, with an incidence of only 5%, and are predominantly located at the junction of the hard and soft palate [3,4]. Most pediatric salivary gland tumors are benign, arising from mesenchymal, epithelial (e.g., pleomorphic adenoma), and neural derivatives. Malignancies, on the other hand, come from epithelial (carcinomas), mesenchymal (sarcomas), and hematolymphoid derivatives. Excluding vascular lesions, pediatric salivary gland neoplasms have a higher malignant rate of 50% compared to 15–25% in adults [5]. Most salivary malignancies (over 80%) occur in the parotid glands. In contrast to adults, children and adolescents with salivary gland malignancies (SGMs) exhibit a narrower histologic spectrum, which is characterized by distinct variations in biological behavior. Epithelial malignancies are more common in children than adults, with mucoepidermoid carcinoma and acinic cell carcinoma as the most common malignant solid tumors in the parotid gland. Mucoepidermoid carcinoma accounts for 33.3% of all salivary malignancies and over 50% of malignant parotid tumors. The diagnosis of SGMs is often delayed for a period of 12 to 24 months due to their characteristic slow growth and lack of symptoms [5,6,7]. Although metastases occurring at the time of diagnosis are rare, the lungs are the most commonly affected site when they do occur [8]. Factors such as an age at diagnosis of under 10 years, previous radiation exposure, rapid growth, presence of pain, and facial weakness are associated with poorer prognosis [5,9,10,11,12]. Despite the identification of potential risk factors, such as a history of radiation exposure [13], the exact etiology of SGM remains elusive. While instances of familial clustering have been observed, a definitive genetic predisposition for salivary gland tumors has not been identified. It is suggested that the development of SGM may arise from the combined influence of genetic and environmental factors [14,15,16,17,18,19].

Additionally, the incidence of SGM in children is lower than in adults, resulting in a limited pool of available data. Therefore, a comprehensive understanding of the epidemiology and potential prognostic factors is crucial for optimizing patient care. Salivary gland malignancies that occur in adults and children have similar histological characteristics, but they exhibit differences in prognostic factors [20], which necessitate varying therapeutic strategies. The primary treatment for salivary gland cancer is typically surgery, but for children, a more conservative approach may be considered to preserve as much gland function as possible. Chemotherapy is not typically used as the primary treatment for salivary gland cancer, but for children, it may be combined with surgery and radiation therapy based on the type and stage of cancer.

Population-based analyses provide valuable epidemiological data and may reveal potential new prognostic factors impacting overall treatment strategies and outcomes. A previous report assessed pediatric cases in the National Cancer Database (NCDB) [9] diagnosed from 2004 to 2013 and determined surgery plus adjuvant radiation is associated with improved overall survival. We anticipate new epidemiological trends exist in more recent years and believe a larger cohort could reveal new prognostic factors.

The purpose of this study is to investigate the demographics and general characteristics of major SGM among individuals aged 21 years and below, with a specific emphasis on identifying the most prevalent histopathological types of salivary gland tumors. This study aims to explore the relationship between tumor types and age, gender, and race, as well as the differences in stage of presentation and histology across age groups and races. Additionally, this study aims to determine any factors that may affect survival rates at 5 and 10 years.

## 2. Materials and Methods

### 2.1. Data Source and Patient Selection

The data used in this study were solely deidentified. As a result, our quaternary center’s Institutional Review Boards determined that this study was exempt from review (University of Tennessee Health Science Center # 21-08528-XM, St Jude Children’s Research Hospital #22-1058). Deidentified data from 2004 to 2018 were acquired from the NCDB [21], a national comprehensive oncology surveillance program established with the collaborative effort from the American Cancer Society and the American College of Surgeons (ACoS) Commission on Cancer (CoC). Patients 21 years of age and under who presented with a diagnosis of major salivary gland tumor were identified by matching the site salivary gland as defined using International Classification of Disease for Oncology Third Edition (ICD-O-3) codes [22]. In the present study, we limited our topic to the parotid gland (C079), submandibular gland (C080), sublingual gland (C081), and any malignant neoplasm of the major salivary gland, unspecified (C089).

### 2.2. Variables

Margins were considered positive if microscopic or macroscopic residual tumors were reported. Surgical variables which included “4 or more lymph nodes” were classified as neck dissection and “less than 4 lymph nodes” were classified as cervical node sampling. Tumors poorly and undifferentiated were classified as high-grade and well- and moderately differentiated malignancies were classified as “low-grade.” American Joint Committee on Cancer (AJCC) stage I and II tumors were categorized as “low-stage”, and stages III and IV were “high-stage”.

### 2.3. Statistical Analysis

A chi-squared test (or Fisher’s exact test if any expected count was <5) was used for testing the association between two factors. A Cochran–Mantel–Haenszel test was used for testing the association with subgroups. Continuous data were expressed as means ± SD, with a Wilcoxon rank-sum test used for testing differences between two groups, and a Kruskal–Wallis test used for testing differences among three or more groups.

We used Kaplan–Meier curves to report the months from diagnosis to death according to histology and other stratification criteria. A logrank test was used for comparing survival curves. Five-year survival rate was calculated and compared between groups using a chi-squared test. All analyses were performed using R software (R: A Language and Environment for Statistical Computing, 4.2.0, R Foundation for Statistical Computing, Vienna, Austria).

## 3. Results

### 3.1. Demographics and Tumor General Characteristics

The database search identified 972 patients aged 21 years and under, 5 of which were benign (histologic codes 8000, 8830, 9260, 9080) and subsequently excluded. Of the final 967 cases of major SGMs (572 females, median age = 17 years, IQR = 5), 55 cases were reported as secondary tumors. Table 1 shows the demographic and tumor characteristics.

### 3.2. Age, Gender, and Racial Differences in Histopathologic Characteristics of Malignancies

Mucoepidermoid carcinoma (MEC, 41.3%) and acinic cell adenocarcinoma (ACA, 33.6%) were the most common SGM, followed by adenoid cystic carcinoma (ACC, 7.4%). Parotid tumors accounted for 86% of cases and showed similar histologic distributions, with MEC most common (41.8%), followed by ACA (38.2%) and ACC (5.2%). While MEC was the most prevalent histology in any major salivary gland location, ACC was the second most common histological type encountered in the submandibular gland (26.5% of cases).

This study conducted subgroup analyses to explore potential differences in histology based on age, gender, and race. The results revealed that the most frequent pathologies observed in the age group of 0 to 5 years were MEC and myoepithelial carcinoma/sarcoma/rhabdomyosarcoma (MC/sarcoma/RMS), which accounted for 33.3% of cases each (N = 8). Additionally, squamous cell carcinoma (SCC) was reported as the third most common histology in this age group, representing 8.3% of cases (*N* = 2).

The proportion of parotid space MC/sarcoma/RMS tumors varied with age. MC/sarcoma/RMS represented 14.3% of cases in the 6–10 age group, 2.1% in the 10–15 age group, and 3.3% in the 16–21 age group. MEC was the most frequent tumor encountered after 5 years of age, representing 56% of the cases in the 6–10 age group, 41.8% in the 11–15 age group, and 39.2% in the 16–21 age group. The proportion of ACA varied according to age: 4.2% in the 0–5 age group, 14.3% in the 6–10 age group, 40% in the 11–15 age group, and 34.5% in the 16–21 age group.

Overall, MEC (38.8%) was the most common malignancy of the parotid and submandibular glands, followed by ACA (26.5%), MC/sarcoma/RMS (7.1%), and SCC (5.1%). A similar trend was observed for sublingual gland tumors, with MEC (60%), MC/sarcoma/RMS (20%), and ACA (20%) representing the most common histopathological findings.

**Figure 1:**
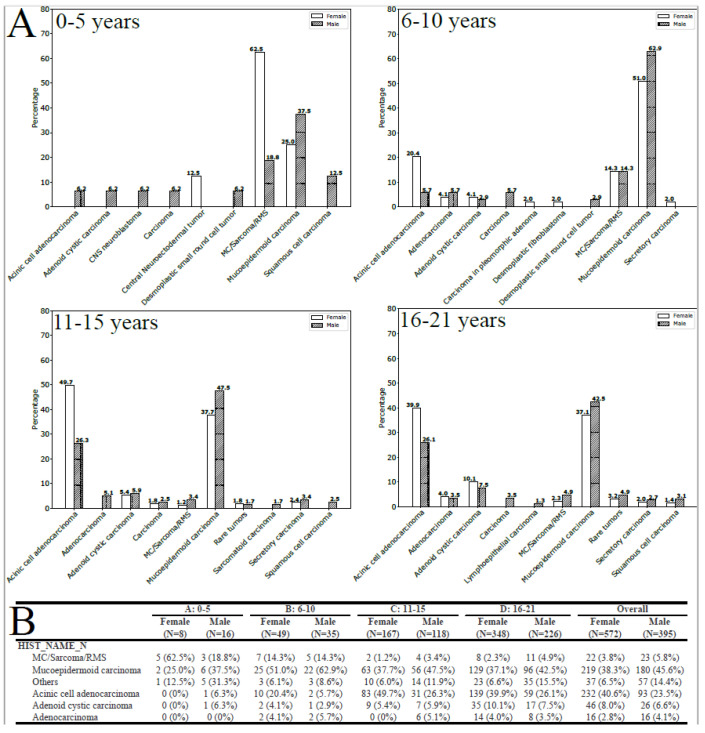
(**A**) Histology by gender and age-group (entire population, all locations). (**B**) Tumors with a frequency inferior to 1% were grouped as rare tumors.

The results of the subgroup analyses demonstrated statistically significant relationships between histology, age group, and gender (*p* < 0.001, as illustrated in Figure 1A,B). In the 0–5 age group, the most commonly encountered tumors in girls were MC/sarcoma/RMS tumors (62.5%), whereas in boys, the most frequently observed tumors were MEC (37.5%). In contrast, MEC was the predominant histology in patients aged six and above, with a higher incidence in boys, while ACA mainly affected girls (as depicted in Figure 1B).

**Figure 2:**
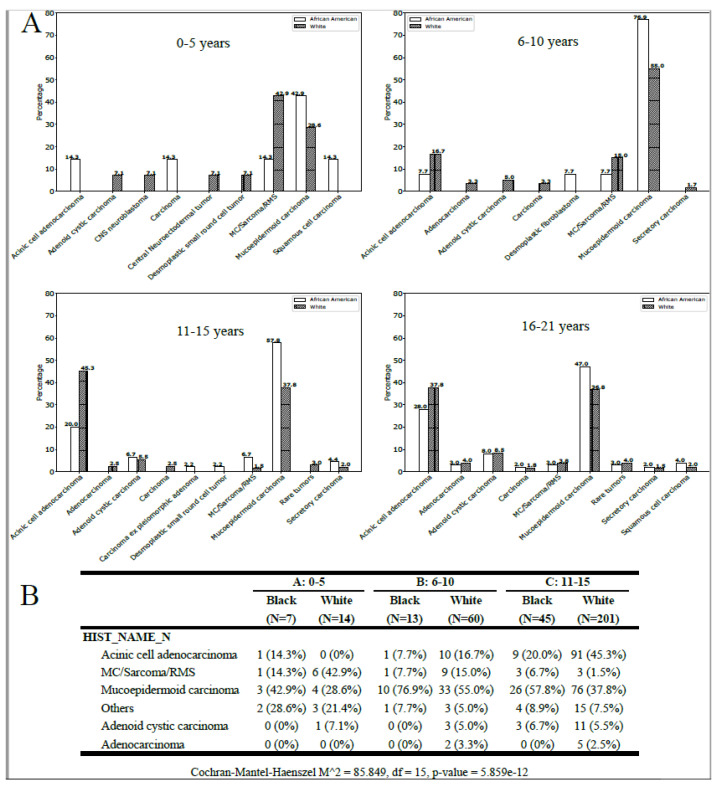
(**A**) Histology for the African American and White pediatric population by age-group (all locations). (**B**) Tumors with a frequency inferior to 1% were grouped as rare tumors.

The statistical analysis revealed a significant correlation between histology, age group, and race (*p* < 0.001; Figure 2B). Among patients aged 0–5 years, MC/sarcoma/RMS tumors were more frequently observed in White patients, while MEC tumors were more common in African American patients across all age groups (Figure 2). The incidence of ACC was extremely low in the 0–5-year-old White population but increased with age (Figure 2A,B). In the 16–21-year-old White population, ACA was the most commonly observed parotid malignancy, while MEC was more frequent in the African American population (Figure 2B).

### 3.3. Differences in Stage of Presentation and Histology by Age Groups and Race

**Figure 3:**
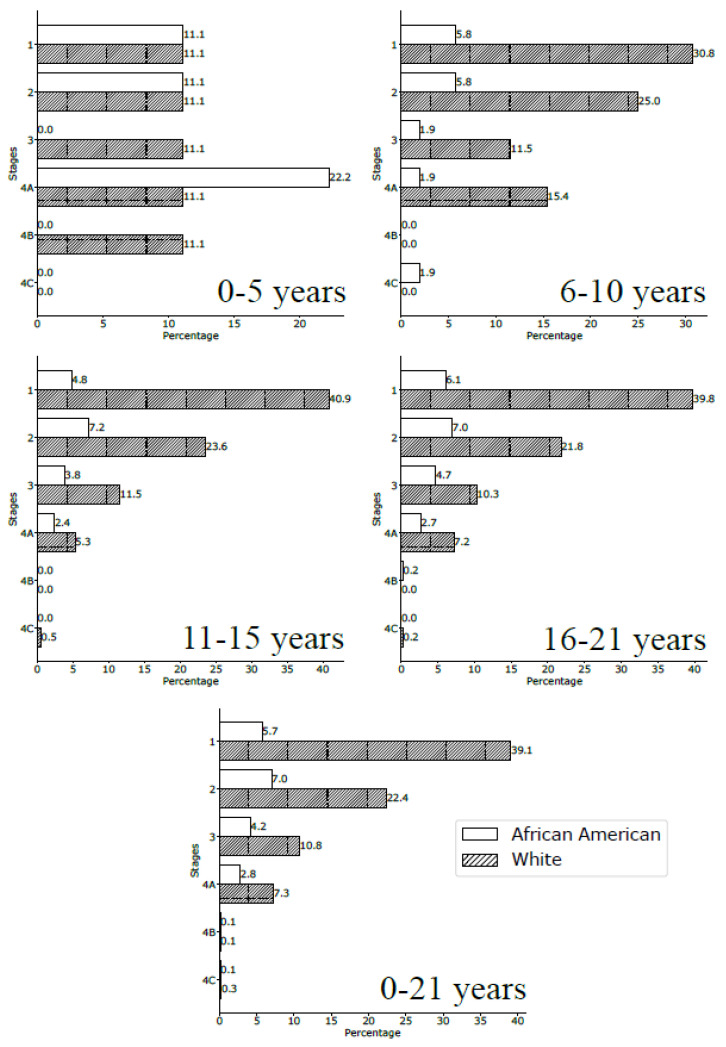
Stages at diagnosis for the African American and White pediatric population by age-group (all locations).

We observed a significant association between tumor stage and age group (*p* < 0.001). Overall, stage I malignancies were the most often encountered (39.3% of cases); however, age and race differences were noted. In the 0–5-year-old population, tumors presented more often as stage IV (16.7% of cases) and were represented by MEC in two cases (one African American child and one White child), SCC in one patient, and MC/sarcoma/RMS in another case (Figure 3). Children older than 5 years presented more commonly with stage I tumors. African American children presented slightly more often with a stage II malignancy and White children presented with stage I (Figure 3).

ACA and MEC were the most encountered histology in stage I parotid malignancies, with ACA more prevalent than MEC in all age groups except for ages 6–10 years, for whom the MEC subtype prevailed. Regardless of the age group, ACA was the most prevalent subtype in stage II parotid malignancies in 6-year-old children and older. Under 6 years old, only one tumor was recorded as a “carcinoma.”

MEC was the most common subtype in stage III parotid malignancies (60%), while ACA and ACC were the second and third (16% and 9%), respectively. Stage IV parotid malignancies were mostly represented by MEC with 56.7% of cases. Overall, ACA, MEC, and ACC most often presented as stage I parotid malignancies.

Regional lymphatic metastases were present in 11.15% of the 0–21-year-old patients (107 of 959 cases), with a higher prevalence in the 6–10-year-olds (18.1%) and lower prevalence in 0–5-year-olds (8.3%, *p* = 0.067). Local lymphatic metastases varied with tumor location: 9.9% (82 of 828 cases) of parotid tumors, 18.8% (18 of 96) of submandibular neoplasms, and 40% (2 of 5) of sublingual malignancies, *p* = 0.007.

The majority of tumors were classified as low-grade with 282 “well-differentiated” cases and 202 “intermediate” cases. High-grade tumors were observed and categorized as “poorly differentiated” in 60 cases and “undifferentiated/anaplastic” in 28 cases. High-grade tumors were significantly associated with advanced T-stage and positive neck disease (*p* < 0.001).

### 3.4. Margin Status

Margin status was available for 874 cases. Margins were positive in 241 cases (24.9%), of which 11.2% (27 of 241) received chemotherapy and 63.1% (152 of 241) underwent radiation. We observed a significant association between positive margin status and the following variables: chemotherapy (*p* = 0.002), radiotherapy *(p <* 0.001), and regional positive nodes *(p* = 0.001).

### 3.5. Tumor Features and Therapies

Therapies varied by tumor location and histology; however, surgery was the primary treatment. We observed a significant association between tumor location and therapies. Adjuvant radiation was reported in 37.3% of parotid malignancy cases (311 of 833), 49% of patients with a submandibular gland neoplasm (48 of 98), and 20% of the sublingual tumors (1 of 5), *p* > 0.05. Radiation was administered to most of the patients diagnosed with ACC (70.8%) with associated chemotherapy in 8.3% of cases (Table 2). Radiation was administered in 55.6% of the MC/sarcoma/RMS, 50% of the adenocarcinoma, 43.4% of the MEC, and 19.1% of the acinic cell adenocarcinomas. With regard to radiation therapy and tumor stage, 34.1% of stage I tumors, 51.9% of stage II tumors, 60.8% of stage III tumors, and 60.3% of stage IVA tumors received radiation. Chemotherapy was considered for 6.5% of patients with a parotid neoplasm (53 of 833 cases), 20.4% of submandibular tumors (20 of 98), and 40% of sublingual malignancies *(p <* 0.001). Chemotherapy was considered in 55.6% of MC/sarcoma/RMS, 8.3% of ACC, and 6.3% of MEC, *p* < 0.001.

Therapies varied with tumor stage. Lower stages were less likely to receive chemotherapy and/or radiation but were more likely to undergo surgical resection alone (*p* < 0.001). A univariate association test revealed a significant association between chemotherapy and the older age group, higher tumor stage and grade, and the presence of local lymph node metastases (all *p*-values < 0.001). Radiation was associated with grade, histology (MC/RMS/sarcoma), positive margins, and stage with significant odd ratios (all *p*-values < 0.001). Similarly, chemotherapy and radiation were significantly associated with tumor type, stages, and grades: the less differentiated, the higher the likelihood for chemotherapy and/or radiotherapy *(p* < 0.001).

### 3.6. Age and Histological Considerations for Radiation

There were significant age distributional differences in radiation treatment, with a median age of 17 years in the radiotherapy group versus 16 years of age in the non-radiotherapy group (*p* = 0.0375). The subgroup analyses revealed significant distributional age differences. Patients presenting with MEC were significantly older in the radiation group than in the non-radiated group (median age 17 and 16 years, respectively, *p* = 0.0491). Distributional differences were observed for stage I (*p* = 0.007) and II (*p* = 0.0491) tumors, for which patients who received radiotherapy were significantly older than patients in the non-radiation group.

### 3.7. 5-Year Survival Analyses

**Figure 4:**
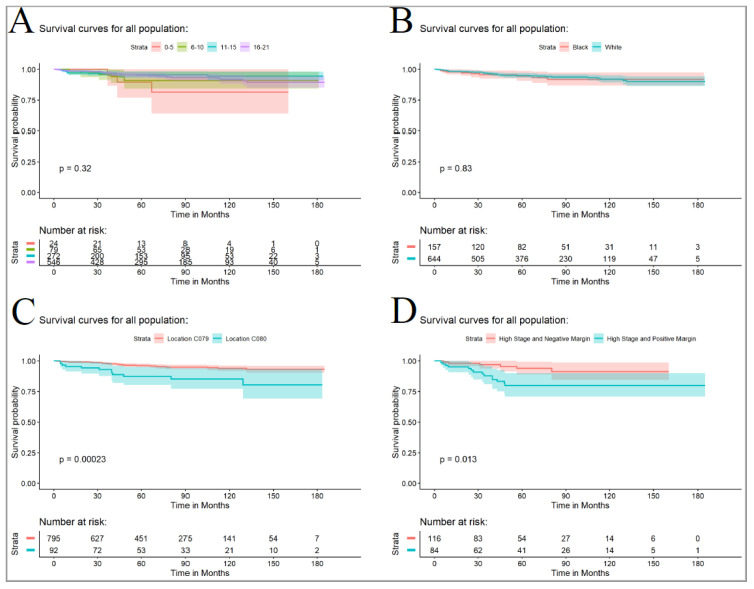
(**A**) Comparison of disease-free survival by age group. (**B**) Comparison of disease-free survival for the African American and White pediatric population. (**C**) Comparison of disease-free survival for parotid (C079) and submandibular tumors (C080). (**D**) Comparison of disease-free survival for tumor with high stage tumors with positive and negative margins. The reported P-values are from logrank analyses (entire curves comparison).

The overall 5-year survival rate was 94.9% (95% CI—93% to 96%) with no significant difference between the lowest and highest rates (chi-squared test; *p* = 0.331). The 0 to 5-year-old group had the lowest survival rate of 90% (95% CI—76% to 100%), while the 11–15-year-old group had the highest survival rate of 96% (95% CI—93% to 98%), as shown in Figure 4A. There were no significant racial differences (*p* = 0.816), as shown in Figure 4B. Histology was found to significantly affect the 5-year survival rate (*p* = 0.003), with a lower survival rate observed for RMS (78%, 95% CI—66% to 93%) compared to epithelial salivary gland neoplasms (96%, 95% CI—94% to 98%). Tumor location was also found to significantly affect the 5-year survival rate (*p* = 0.009), with parotid malignancies having a higher survival rate (96%, 95% CI—95% to 98%) compared to submandibular neoplasms (87%, 95% CI—80% to 95%), as shown in Figure 4C. Margin status was found to be significantly associated with 5-year survival in high-stage tumors (*p* = 0.003), with negative margins showing an observed survival rate of 94% (95% CI—89% to 99%) compared to positive margins, which had a survival rate of 80% (95% CI—71% to 90%), as shown in Figure 4D.

## 4. Discussion

Malignancies of the major salivary glands (MSGs) are rare in children, and investigating epidemiologic and prognostic factors are key to improving survival. The majority of pediatric MSG malignancies occur in the parotid gland [23] and are of epithelial origin, with a predominance of MEC and ACA [1,3,4,10,11]. While some reports favor MEC as the most common carcinoma [2,23,24,25,26], others support a predominance of ACA or nearly equal frequencies of ACA and MEC [27,28,29,30,31]. The epidemiology of pediatric salivary gland tumors varies by geographic location [32,33], but our findings also show variation by age group, race, and gender. We confirmed an overall female predominance [4,34,35,36], and MSG tumors tend to be rarer during the first decade [4], with 88.9% of cases reported in the 11–21-year-old population. Contrary to a previous report [2], MEC dominated the submandibular location with ACC as the second most frequent malignancy. We observed the pediatric ratio of parotid-to-submandibular gland malignancies to be somewhat different from the adult population. We found a greater rate of pediatric MSG tumors (86.1%) located in the parotid gland. In the 0–5-year-old population, MC/sarcoma/RMS and MEC were observed to be the most common MSG tumors. These tumor classes were equally represented but with a different sex ratio: a female predominance for MC/sarcoma/RMS. ACA was the predominant histology encountered in females in the 11–21-year-old group, whereas MEC dominated in males. Racial differences were observed in the 0–5-year group with a notable predominance of White children presenting with MC/sarcoma/RMS pathology and African Americans presenting with a MEC. ACA tumors were most prevalent in females, as suggested in other adult series [37,38,39], and MEC was more frequent in males.

In accordance with previous studies [4,40], MSG malignancies were most commonly low-grade and presented as stage I. However, a predominance was observed in the 0–5-year-old group for stage IV MSG tumors, which were MEC in 50% of the cases. The prevalence of regional positive lymph node metastases observed in our study was quite similar to the 10–17% reported in the literature [7,23].

As observed in the present report, initial surgical resection remained the mainstay of treatment [4,41]. Our results validate ACC of MSGs to behave similarly to other ACC, with resection and radiation as the mainstay of treatment. Moreover, carcinoma/sarcoma/rhabdomyosarcoma of the salivary gland was treated similarly to other anatomic locations, with resection and chemoradiation [2,42]. The utilization of chemotherapy in the management of salivary gland tumors remains a topic of debate. In the present report, univariate association tests demonstrated a significant correlation between chemotherapy and the older age group, higher tumor stage and grade, and the presence of local lymph node metastases. However, very few studies have investigated the efficacy of systemic therapy, and those that do exist primarily involve adult populations [43,44]. As of now, there is no consensus on the optimal chemotherapy regimens for salivary gland carcinomas. In the literature, chemotherapy is typically reserved for palliative purposes, targeting recurrent and/or metastatic disease that is not amenable to further surgery or radiation treatment. Despite the inclusion of RMS/myoepithelial tumors in our present analysis, a greater number of pediatric cases were considered for radiation, suggesting a change in care strategy. Radiotherapy was traditionally reserved for patients with adverse histological features or recurrent disease [45,46]. The current utilization of radiation in children is still debated for intermediate- and low-grade tumors. However, our findings suggest a growing presence of radiotherapy in the care strategy, with radiation rates exceeding those previously reported [9,47] but still marginally inferior to what is observed in the adult population. This trend may be partially due to the increased use of proton beam therapy for pediatric head and neck malignancies with reportedly reduced radiation morbidities [48,49,50].

Overall, patients with higher tumor grade and/or stage received adjuvant therapies. Our study has corroborated previous reports that radiation therapy is more frequently recommended after surgical treatment for ACA and ACC [47] than for MEC [47]. While long-term survivorship rates in this study showed less radiotherapy-related morbidity than commonly observed in the adult population [23], the impact of radiation and its role in the pediatric population for the treatment of MSG malignancies is only partially defined. Furthermore, previous reports suggest a little role of radiation for patients with MEC [7]. Our findings corroborate a recently suggested trend [1] showing an increased consideration for radiotherapy in MEC with 43.5% of the present MEC cohort treated with adjuvant radiation. This trend is apparent for low-stage tumors as well. Overall, we observed a higher rate of patients considered for radiation than in previous studies [23,51], but age-related differences were noted for low-stage tumors (I and II) and MEC, for which radiotherapy was considered for older patients. In light of these observations, it is imperative to contextualize the finding that, in the present report, radiation therapy did not exhibit a significant impact on overall survival in pediatric patients, as compared to previous publications [7]. Given the potential sequelae associated with radiotherapy [7,8,23,52], it is crucial to better understand and assess the role of radiation in this vulnerable population. While there are currently no established treatment guidelines in the United States specifically for managing pediatric salivary gland cancer, much of the care strategy is derived from recommendations provided for the adult population. Recently, the European Cooperative Study Group for Pediatric Rare Tumors (EXPeRT) in collaboration with the EU-funded PARTNER project (Paediatric Rare Tumors Network European Registry) introduced consensus recommendations for the diagnosis and treatment of pediatric salivary gland carcinomas. These recommendations were developed based on a Consensus Conference and are outlined in the Standard Care Strategy Flowchart proposed by the Pediatric Rare Tumor Network [52]. Based on their research, the group suggests that chemotherapy and/or radiotherapy are typically the initial treatment options to be considered for cases of metastatic salivary gland carcinoma and/or unresectable tumors, and surgery with be delayed. For all other cases, surgical intervention is recommended as the first-line treatment. The decision to proceed with radiotherapy before a second-look surgery is based on the quality of the initial surgical resection. Moreover, in cases outside of this context, radiotherapy is considered for high stages irrespective of the lymph node status. Additionally, radiotherapy to the lymph nodes is recommended for high-grade tumors, the involvement of multiple nodes, and the presence of extranodal extension, with a specific emphasis on administering radiotherapy to both the primary tumor and the lymph nodes [52]. Nevertheless, there is a pressing need for global consensus and the establishment of large cohort databases to ensure greater standardization and improved evaluation of the care strategy.

The overall survival (OS) was similar to the previously reported rate of 93–95% [8,23] and was found to be superior to the OS of adult populations. Similarly, the observed 5-year OS for MEC was higher in our pediatric cohort than in adults (68–75%) [53,54]. This may be explained by an earlier stage at presentation with a lower rate of local metastases [8]. Previous studies suggested OS differences between cohorts aged 15 years and older versus those under 15 years [47]; however, our findings suggest the presence of more subtle trends in the group of children under 15 years old. The location of the MSG malignancy matters. The 5-year OS rates were significantly worse for submandibular malignancies, regardless of the patient age group. Survival for rhabdomyosarcoma was poorer than that for epithelial salivary gland neoplasms and local, nondeforming resection followed by chemotherapy, and radiotherapy was undeniably the most commonly recommended treatment regimen for RMS [2,55].

In the present cohort, our observations affirm the criticality of surgical margins in patient outcomes. This corroborates earlier findings in the adult population, where research has demonstrated that achieving negative margins leads to improved disease-free survival rates and reduced instances of local recurrence [53]. It is important to note that attaining negative margins poses greater challenges in pediatric cases. Factors such as nerve involvement or circumferential tumor presence in children further complicate the delicate balance between ensuring local control and deciding on adjuvant radiotherapy, which may have long-term implications. With the ongoing development and wider implementation of proton therapies, this intricate dilemma is anticipated to evolve. Nevertheless, recent literature strongly emphasizes the use of stringent long-term follow-up protocols that extend beyond a five-year timeframe. Such an approach ensures thorough monitoring of potential late effects and enables prompt intervention or management of any emerging complications that may arise [7,52].

It should be noted that the NCDB is capturing about 70% of the malignancies in the United States, which may affect the conclusion regarding frequencies and incidences. Perineural and vascular invasion as well as genetic and molecular findings were not captured in the present study.

However, population-based analyses provide valuable clues for rare diseases. This study provides new insight into age, racial, and gender differences in MSG tumors and also illuminates evolving therapeutic strategies with greater importance given to radiation.

## 5. Conclusions

This study provides valuable insights into the demographics and histopathologic characteristics of major SGM in children and young adults. Our findings shed light on the variations in histology by age, gender, and race, and underscore the importance of incorporating demographic and histopathologic characteristics into the diagnosis and management of these tumors, particularly in the context of personalized medicine. To improve standardization and further evaluate care strategies, there is a need for global consensus and the establishment of large cohort databases.

## Figures and Tables

**Table 1:** Demographics and tumor characteristics.

Demographics and Tumor Characteristics		*N*	%
Sex	Female	572	59.1
	Male	395	40.8
Race	White	675	69.8
	African American	165	17.1
	Asian	54	5.6
	American Indian, Aleutian, or Eskimo	10	1
	Pacific islander	11	1.1
	Other/Unknown	52	5.4
Age distribution (years)	0–5	24	2.5
	6–10	84	8.7
	11–15	285	29.5
	16–21	574	59.4
Location	Parotid gland	833	86.1
	Submandibular gland	98	10.1
	Sublingual gland	5	0.5
	Unspecified	31	3.2
Histology	Mucoepidermoid carcinoma	399	41.3
	Acinic cell adenocarcinoma	325	33.6
	Adenoid cystic carcinoma	72	7.4
	Sarcoma, MC & Rhabdomyosarcoma	45	4.7
	Adenocarcinoma	32	3.3
	Others	94	9.7

**Table 2:** Treatment characteristics by histology.

	Acinic Cell Adenocarcinoma (*N* = 325)	Adenocarcinoma (*N* = 32)	Adenoid Cystic Carcinoma (*N* = 72)	MC/Sarcoma/RMS (*N* = 45)	Mucoepidermoid Carcinoma (*N* = 399)	Others (*N* = 94)	*p*-Value
Chemotherapy							
No	325 (100%)	29 (90.6%)	66 (91.7%)	20 (44.4%)	372 (93.7%)	69 (73.4%)	<0.001
Yes	0 (0%)	3 (9.4%)	6 (8.3%)	25 (55.6%)	25 (6.3%)	25 (26.6%)	
Surg_prim_site							
No	7 (2.2%)	1 (3.1%)	2 (2.8%)	11 (24.4%)	4 (1.0%)	11 (11.7%)	<0.001
Yes	318 (97.8%)	31 (96.9%)	70 (97.2%)	34 (75.6%)	395 (99.0%)	83 (88.3%)	
Radiation							
No	263 (80.9%)	16 (50.0%)	21 (29.2%)	20 (44.4%)	226 (56.6%)	48 (51.1%)	<0.001
Yes	62 (19.1%)	16 (50.0%)	51 (70.8%)	25 (55.6%)	173 (43.4%)	46 (48.9%)	

## Data Availability

Data from the NCDB can be obtained upon request to the NCDB.

## References

[1] Janz T.A., Camilon P.R., Nguyen S.A., Levi J.R., Lentsch E.J. (2018). Has the management of pediatric mucoepidermoid carcinoma of the parotid gland changed?. Laryngoscope.

[2] Shapiro N.L., Bhattacharyya N. (2006). Clinical characteristics and survival for major salivary gland malignancies in children. Otolaryngol. Head Neck Surg..

[3] Zamani M., Grønhøj C., Schmidt Jensen J., von Buchwald C., Charabi B.W., Hjuler T. (2019). Survival and characteristics of pediatric salivary gland cancer: A systematic review and meta-analysis. Pediatr. Blood Cancer.

[4] Louredo B.V.R., Santos-Silva A.R., Vargas P.A., Lopes M.A., Martins M.D., Guerra E.N.d.S., Ribeiro A.C.P., Brandão T.B., Mendonça R.M.H., Kowalski L.P. (2021). Clinicopathological analysis and survival outcomes of primary salivary gland tumors in pediatric patients: A systematic review. J. Oral Pathol. Med..

[5] Gontarz M., Wyszyńska-Pawelec G., Zapała J. (2018). Primary epithelial salivary gland tumours in children and adolescents. Int. J. Oral Maxillofac. Surg..

[6] Yoshida E.J., García J., Eisele D.W., Chen A.M. (2014). Salivary gland malignancies in children. Int. J. Pediatr. Otorhinolaryngol..

[7] Thariat J., Vedrine P.O., Temam S., Ali A.M., Orbach D., Odin G., Makeieff M., Nicollas R., Penicaud M., Toussaint B. (2013). The role of radiation therapy in pediatric mucoepidermoid carcinomas of the salivary glands. J. Pediatr..

[8] Sultan I., Rodriguez–Galindo C., Al-Sharabati S., Guzzo M., Casanova M., Ferrari A. (2011). Salivary gland carcinomas in children and adolescents: A population-based study, with comparison to adult cases. Head Neck.

[9] Morse E., Fujiwara R.J.T., Husain Z., Judson B., Mehra S. (2018). Pediatric Salivary Cancer: Epidemiology, Treatment Trends, and Association of Treatment Modality with Survival. Otolaryngol. Head Neck Surg..

[10] Lennon P., Silvera V.M., Perez-Atayde A., Cunningham M.J., Rahbar R. (2015). Disorders and tumors of the salivary glands in children. Otolaryngol. Clin. North Am..

[11] Luna M.A., Batsakis J.G., El-Naggar A.K. (1991). Salivary gland tumors in children. Ann. Otol. Rhinol. Laryngol..

[12] Bradley P., McClelland L., Mehta D. (2007). Paediatric salivary gland epithelial neoplasms. ORL J. Otorhinolaryngol. Relat. Spec..

[13] Radoï L., Barul C., Menvielle G., Carton M., Matrat M., Sanchez M., Pilorget C., Velten M., Stücker I., Luce D. (2018). Risk factors for salivary gland cancers in France: Results from a case-control study, the ICARE study. Oral Oncol..

[14] Albeck H., Bentzen J., Ockelmann H.H., Nielsen N.H., Bretlau P., Hansen H.S. (1993). Familial clusters of nasopharyngeal carcinoma and salivary gland carcinomas in Greenland natives. Cancer.

[15] Merrick Y., Albeck H., Nielsen N.H., Hansen H.S. (1986). Familial clustering of salivary gland carcinoma in Greenland. Cancer.

[16] Depowski P.L., Setzen G., Chui A., Koltai P.J., Dollar J., Ross J.S. (1999). Familial occurrence of acinic cell carcinoma of the parotid gland. Arch. Pathol. Lab. Med..

[17] Aro K., Klockars T., Leivo I., Mäkitie A. (2014). Familial Predisposition for Salivary Gland Cancer in Finland. Clin. Med. Insights Ear. Nose Throat..

[18] Delides A., Velegrakis G., Kontogeorgos G., Karagianni E., Nakas D., Helidonis E. (2005). Familial bilateral acinic cell carcinoma of the parotid synchronous with pituitary adenoma: Case report. Head Neck.

[19] Horn-Ross P.L., Ljung B.M., Morrow M. (1997). Environmental factors and the risk of salivary gland cancer. Epidemiology.

[20] Acharya S., Sinard R.N., Rangel G., Rastatter J.C., Sheyn A. (2021). Rethinking the Definition of High Risk in Pediatric Salivary Gland Carcinoma. Otolaryngol. Head Neck Surg..

[21] Bilimoria K.Y., Stewart A.K., Winchester D.P., Ko C.Y. (2008). The National Cancer Data Base: A Powerful Initiative to Improve Cancer Care in the United States. Ann. Surg. Oncol..

[22] International Classification of Diseases for Oncology, 3rd Edition (ICD-O-3). https://www.who.int/standards/classifications/other-classifications/international-classification-of-diseases-for-oncology.

[23] Kupferman M.E., de la Garza G.O., Santillan A.A., Williams M.D., Varghese B.T., Huh W., Roberts D., Weber R.S. (2010). Outcomes of Pediatric Patients with Malignancies of the Major Salivary Glands. Ann. Surg. Oncol..

[24] Jones A.V., Craig G.T., Speight P.M., Franklin C.D. (2008). The range and demographics of salivary gland tumours diagnosed in a UK population. Oral Oncol..

[25] Spiro R.H. (1986). Salivary neoplasms: Overview of a 35-year experience with 2807 patients. Head Neck Surg..

[26] Spitz M.R., Batsakis J.G. (1984). Major salivary gland carcinoma. Descriptive epidemiology and survival of 498 patients. Arch. Otolaryngol..

[27] Goode R.K., Auclair P.L., Ellis G.L. (1998). Mucoepidermoid carcinoma of the major salivary glands: Clinical and histopathologic analysis of 234 cases with evaluation of grading criteria. Cancer.

[28] Luukkaa H., Klemi P., Leivo I., Koivunen P., Laranne J., Mäkitie A., Virtaniemi J., Hinkka S., Grénman R. (2005). Salivary gland cancer in Finland 1991–96: An evaluation of 237 cases. Acta Otolaryngol..

[29] Ostman J., Anneroth G., Gustafsson H., Tavelin B. (1997). Malignant salivary gland tumours in Sweden 1960-1989--an epidemiological study. Oral Oncol..

[30] Wahlberg P., Anderson H., Biörklund A., Möller T., Perfekt R. (2002). Carcinoma of the parotid and submandibular glands--a study of survival in 2465 patients. Oral Oncol..

[31] Onyango J.F., Awange D.O., Muthamia J.M., Muga B.I. (1992). Salivary gland tumours in Kenya. East Afr. Med. J..

[32] Deng R., Huang X., Hao J., Ding J., Hu Q. (2013). Salivary gland neoplasms in children. J. Craniofac. Surg..

[33] da Silva L.P., Serpa M.S., Viveiros S.K., Sena D.A.C., Pinho R.F.D.C., Guimarães L.D.D.A., Andrade E.S.D.S., Pereira J.R.D., da Silveira M.M.F., Sobral A.P.V. (2018). Salivary gland tumors in a Brazilian population: A 20-year retrospective and multicentric study of 2292 cases. J. Craniomaxillofac. Surg..

[34] Liu B., Liu J.Y., Zhang W.F., Jia J. (2012). Pediatric parotid tumors: Clinical review of 24 cases in a Chinese population. Int. J. Pediatr. Otorhinolaryngol..

[35] Kessler A., Handler S.D. (1994). Salivary gland neoplasms in children: A 10-year survey at the Children’s Hospital of Philadelphia. Int. J. Pediatr. Otorhinolaryngol..

[36] Laikui L., Hongwei L., Hongbing J., Zhixiu H. (2008). Epithelial salivary gland tumors of children and adolescents in west China population: A clinicopathologic study of 79 cases. J. Oral Pathol. Med..

[37] Gontarz M., Bargiel J., Gąsiorowski K., Marecik T., Szczurowski P., Zapała J., Wyszyńska-Pawelec G. (2021). Epidemiology of Primary Epithelial Salivary Gland Tumors in Southern Poland—A 26-Year, Clinicopathologic, Retrospective Analysis. J. Clin. Med..

[38] de Ridder M., Balm A.J.M., Smeele L.E., Wouters M.W.J.M., van Dijk B.A.C. (2015). An epidemiological evaluation of salivary gland cancer in the Netherlands (1989–2010). Cancer Epidemiol..

[39] Bjørndal K., Krogdahl A., Therkildsen M.H., Overgaard J., Johansen J., Kristensen C.A., Homøe P., Sørensen C.H., Andersen E., Bundgaard T. (2011). Salivary gland carcinoma in Denmark 1990-2005: A national study of incidence, site and histology. Results of the Danish Head and Neck Cancer Group (DAHANCA). Oral Oncol..

[40] Locati L.D., Collini P., Imbimbo M., Barisella M., Testi M.A., Licitra L., Löning T., Tiemann K., Quattrone P., Bimbatti E. (2017). Immunohistochemical and molecular profile of salivary gland cancer in children. Pediatr. Blood Cancer.

[41] Radomski S., Dermody S., Harley E.H. (2018). Clinical characteristics and outcomes of major salivary gland malignancies in children. Laryngoscope.

[42] Rogers D.A., Rao B.N., Bowman L., Marina N., Fleming I.D., Schropp K.P., Lobe T.E. (1994). Primary malignancy of the salivary gland in children. J. Pediatr. Surg..

[43] Wang X., Luo Y., Li M., Yan H., Sun M., Fan T. (2016). Management of salivary gland carcinomas—A review. Oncotarget.

[44] Laurie S.A., Licitra L. (2006). Systemic therapy in the palliative management of advanced salivary gland cancers. J. Clin. Oncol..

[45] Guzzo M., Ferrari A., Marcon I., Collini P., Gandola L., Pizzi N.R., Casanova M., Mattavelli F., Scaramellini G. (2006). Salivary gland neoplasms in children: The experience of the Istituto Nazionale Tumori of Milan. Pediatr. Blood Cancer.

[46] Bossé J.P., Cloutier D. (1976). Les masses parotidiennes. Can. Fam. Physician.

[47] Allan B.J., Tashiro J., Diaz S., Edens J., Younis R., Thaller S.R. (2013). Malignant tumors of the parotid gland in children: Incidence and outcomes. J. Craniofac. Surg..

[48] Spiotto M.T., McGovern S.L., Gunn G.B., Grosshans D., McAleer M.F., Frank S.J., Paulino A.C. (2021). Proton Radiotherapy to Reduce Late Complications in Childhood Head and Neck Cancers. Int. J. Part Ther..

[49] Grant S.R., Grosshans D.R., Bilton S.D., Garcia J.A., Amin M., Chambers M.S., McGovern S.L., McAleer M.F., Morrison W.H., Huh W.W. (2015). Proton versus conventional radiotherapy for pediatric salivary gland tumors: Acute toxicity and dosimetric characteristics. Radiother. Oncol..

[50] Hanania A.N., Zhang X., Gunn G.B., Rosenthal D.I., Garden A.S., Fuller C.D., Phan J., Reddy J.P., Moreno A., Chronowski G. (2021). Proton Therapy for Major Salivary Gland Cancer: Clinical Outcomes. Int. J. Part. Ther..

[51] Ryan J.T., El-Naggar A.K., Huh W., Hanna E.Y., Weber R.S., Kupferman M.E. (2011). Primacy of surgery in the management of mucoepidermoid carcinoma in children. Head Neck.

[52] Surun A., Schneider D.T., Ferrari A., Stachowicz-Stencel T., Rascon J., Synakiewicz A., Agaimy A., Martinova K., Kachanov D., Roganovic J. (2021). Salivary gland carcinoma in children and adolescents: The EXPeRT/PARTNER diagnosis and treatment recommendations. Pediatr. Blood Cancer.

[53] McHugh C.H., Roberts D.B., El-Naggar A.K., Hanna E.Y., Garden A.S., Kies M.S., Weber R.S., Kupferman M.E. (2012). Prognostic factors in mucoepidermoid carcinoma of the salivary glands. Cancer.

[54] Vander Poorten V.L., Balm A.J., Hilgers F.J., Tan I.B., Keus R.B., Hart A.A. (2000). Stage as major long term outcome predictor in minor salivary gland carcinoma. Cancer.

[55] Rebours C., Couloigner V., Galmiche L., Casiraghi O., Badoual C., Boudjemaa S., Chauvin A., Elmaleh M., Fresneau B., Fasola S. (2017). Pediatric salivary gland carcinomas: Diagnostic and therapeutic management. Laryngoscope.

